# Gold(III) Ions Sorption on Amberlite XAD-16 Impregnated with TBP After Leaching Smart Card Chips

**DOI:** 10.3390/molecules30010151

**Published:** 2025-01-02

**Authors:** Karolina Zinkowska, Zbigniew Hubicki, Grzegorz Wójcik

**Affiliations:** Department of Inorganic Chemistry, Institute of Chemical Sciences, Faculty of Chemistry, Maria Curie-Skłodowska University, Maria Curie-Skłodowska Sq. 2, 20-031 Lublin, Poland; grzegorz.wojcik2@mail.umcs.pl

**Keywords:** sorption, precious metals, impregnation

## Abstract

Owing to the intensive development of electrical and electronic equipment, there is an increasing demand for precious metals, which are often used for its production. Due to their scarce supply, it is important to recover them from secondary sources. A promising way to recover precious metals are impregnated resins. In this research, Amberlite XAD-16 was impregnated with TBP at the weight ratios of 1:2 and 1:3 using the ‘warm impregnation’ method. Studies were carried out on the sorption of Au(III), Pd(II), Pt(IV), and Rh(III) ions from the model chloride solutions as well as the real solution formed after leaching the smart card chips. Only Au(III) ions were efficiently sorbed on the prepared impregnated sorbents. The best results were obtained at 6 M HCl and the sorbent mass: 0.1 g/25 mL. The maximum sorption capacity for the impregnated sorbents was: 147.91 mg/g (ratio 1:2) and 149.66 mg/g (ratio 1:3). Recovery of Au(III) ions from the real leaching solution was: 97.36% and 97.77%, respectively. The Langmuir isotherm was the best-fit model for the experimental results. Thermodynamic studies proved that the investigated sorption process is spontaneous and exothermic. The desorption process can be easily carried out with 1 M HCl/1 M TU.

## 1. Introduction

The last few centuries have experienced extremely rapid technological progress. As an example, the first airplane flight took place in 1903, and already in 1969, a man landed on the moon for the first time [1]. However, with this technological progress, we are not only considering modern special-purpose technologies. Every now and then, new electronic and electrical devices appear on the market that make people’s daily lives easier. Unfortunately, this results in equipment that is still in working order being replaced by new, marginally better devices. This generates a huge amount of waste, called Waste Electrical and Electronic Equipment (WEEE). According to the statistics, in 2010, the amount of electrical and electronic equipment placed on the market was 62 billion kg, while in 2022, it was already 96 billion kg. Therefore, the amount of generated waste has increased from 34 billion kg in 2010 to 62 billion kg in 2022. The Global E-Waste Monitor 2020 reports that in 2019, only 17.4% of the generated e-waste was recycled [2].

According to the latest data reported by the Global E-Waste Monitor 2024, the amount of documented and recycled waste has increased to only 22.3%. WEEE is predicted to increase to 82 billion kilograms in 2030, and thus, much effort should be made to increase its recycling. This is not only important from an environmental point of view, but also from an economic one. WEEE is an excellent secondary source of valuable and critical metals, for example precious metals like gold, platinum, palladium, or rhodium. Unfortunately, despite the recycling of e-waste, a significant proportion of these metals is lost due to inefficient recovery methods [3].

Precious metals are non-renewable raw materials and their natural resources are rapidly depleting as a result of their intensive exploitation in many industries. In electronics, they are often used, among others, to coat contacts and pins in various electronic devices due to their good electrical conductivity, corrosion resistance, and mechanical strength [4]. For instance, gold is used to cover the microprocessor chips in smart cards. There are two types of smart cards: contact and contactless ones. The contact smart cards contain a chip that is coated with gold. The chip can store information that can be read by a reader [5].

As demand for precious metals continues to grow, new and more efficient methods must be sought to recover them from secondary raw materials. The main methods for their recovery are pyrometallurgical, bio-metallurgical, and hydrometallurgical ones [6]. The pyrometallurgical methods involve combustion, smelting, and slag formation. All reactions are conducted at very high temperatures in specially designed furnaces. The disadvantages of these methods are the high energy intensity and the emission of toxic substances [7]. Typically, pyrometallurgical methods are used for wastes that contain larger amounts of precious metals than are contained in WEEE [8]. The bio-metallurgical methods are based on the use of micro-organisms such as bacteria or fungi that are capable of producing compounds, e.g., hydrogen cyanide, which can extract valuable metals from e-waste [9]. In a study by J.K. Pradhan and et al., the recovery of gold from e-waste using *Chromobacterium violaceum* was about 70% [10]. In contrast, in the study by A. Kumar et al. using *Pseudomonas balearica* SAE1, gold recovery under optimal conditions was 68.5% [11]. The bio-metallurgical methods such as bioleaching are environmentally friendly; however, they also have drawbacks, for example, the used bacteria can be sensitive to the toxins produced during the leaching of e-waste, resulting in smaller yields than those of pyrometallurgical or hydrometallurgical methods. In recent years, hydrometallurgical methods have been gaining in popularity for the recovery of precious metals. They are highly efficient and much less costly, as well as more energy-intensive than the pyrometallurgical methods. They exploit leaching solutions, e.g., HCl in the presence of H_2_O_2_, sulfuric acid, and aqua regia, which digest the waste. The dissolved specific metals pass into a solution and then the solution is subjected to separation and purification through techniques such as solvent extraction, ion exchange, adsorption, electrochemical methods, precipitation, or co-precipitation [12]. A.C. Kasper used sodium thiosulfate and ammonium thiosulfate to leach gold from the printed circuit boards from mobile phones. In this research, it was found that gold extraction rates using 0.12 M sodium and ammonium thiosulfate were 70% and 75%, respectively. For comparison: using a cyanide-based extractant which is less environmentally friendly, it was possible to achieve a gold extraction rate of 88%. Gold recovery from the real leaching solution was made using the electrowinning technique at a potential of −500 mV vs. Ag/AgCl. The results show that the fraction of recovered gold was equivalent to 94% [13]. M. A. Dehchenari et al. recovered gold from CPUs of computer circuit boards. Leaching was performed with nitric acid followed by the use of aqua regia. To remove excess of nitric acid, sulfuric acid was added to the solution. Using iron sulphate to precipitate gold, a recovery of 82.3% was obtained [14]. In the study by A. Cieszynska et al., a mixture of Cyphos^®^ IL 101 and toluene was used as the extractant. Solvent extraction of palladium(II) ions was performed from model chloride solutions with different concentrations of hydrochloric acid. An extraction rate of 97% was obtained for the 1 M HCl solution [15]. V. T. Nguyen et al. applied the FeCl_3_/CH_3_CN system in their study for selectively leaching of PGMs from a spent automotive exhaust converter. Using 0.01 mol/L FeCl_3_ in CH_3_CN, palladium was selectively leached. Then, platinum and rhodium were completely leached using 0.3 mol/L FeCl_3_ in CH_3_CN. In this study, Aliquat 336 (trioctylmethylammonium chloride) was used for selective separation of platinum from rhodium [16]. In research by S. Ilyas, the spent diesel oxidation catalyst was leached using HCl/H_2_O_2_. Under the optimized leaching conditions, it was possible to obtain 94% of palladium(II) and 90% of platinum(IV) in the solution. Separation of platinum ions (98%) from the solution proceeded by precipitation with NH_4_Cl, leaving palladium(II) ions in the solution. Palladium(II) ions were then precipitated with NaClO_3_ [17].

Quite common in the separation of metals from aqueous solutions in recent years are SIRs (Solvent Impregnated Resins). The polymeric matrices are impregnated with various extractants selective for specific components in the solution. In contrast to other separation methods, sorption on solvent-impregnated resins is characterized by low reagent consumption, the possibility of regeneration, low energy consumption, selectivity, as well as relatively low price and simplicity of preparation. For this reason, they have a much lower environmental impact compared to the pyrometallurgical methods described earlier [18]. As a result, they find application in the sorption of dyes, phenolic compounds, or metal ions, including precious metal ions [19]. M. Yamada et al. impregnated the macroporous resin Amberlite 7HP with p-tert-butylthiacalix[4/6]arenes and their thiocarbamoyl derivatives. The SIRs prepared in this way were used for the sorption of palladium(II) ions. The study proved that the presence of thiocarbamoyl groups enhanced the sorption of palladium(II) ions, which was as high as 99%. This was due to the formation of stronger complexes with the ions of this metal. In the presence of other elements in the solution, the sorption of palladium(II) ions decreased [20]. R. Navaro et al. used Amberlite XAD-7 matrix impregnated with Cyphos IL-101 (tetradecyl(trihexyl)phosphonium chloride) and ketone. SIR was used in the Pd(II) ions sorption at various HCl concentrations. The best sorption capacity was obtained for the 0.5 M HCl solution and it was equal to 70 mg/g [21].

TBP (tri-butyl phosphate) is used, among others, for metal ions sorption as an extractant or modifier. For instance, in the studies carried out by B. Chen et al., TBP was mixed with N235 (trialkylamine) and diluted with petroleum. Then, Amberlite XAD-16 was impregnated with this mixture and used to sorb vanadium(V) ions. The sorption capacity obtained in these studies was 50.95 mg/g, which is larger than that for SIR prepared without the TBP addition (46.73 mg/g) [22]. N. Sadeghi et al. used the 0.1–0.25 mol/L TBP solution as the organic phase and 3 M HCl as the aqueous phase in solvent extraction of Au(III) ions from model solutions and the real leach solution of copper anode slime. The extraction efficiency of Au(III) ions was over 95%, while the extraction percentage of the other elements ions (so-called impurities) was much lower [23].

This paper presents the studies on the sorption of precious metals on the impregnated sorbents. Amberlite XAD-16 as sorbent and TBP as extractant were used. The impregnation method, which does not require the use of toxic organic solvents, is described in detail in previous papers [24,25,26]. Pure Amberlite XAD-16 was not capable of sorbing precious metal ions, so it was impregnated with TBP, which proved effective in extraction of Au(III) ions. The sorption studies were carried out from the model chloride solutions to select the optimum sorption conditions, followed by the studies in the real solution after leaching gold chips from the smart cards.

## 2. Results

### 2.1. Characterization of the Impregnated Sorbent

#### 2.1.1. Scanning Electron Microscopy Analysis (SEM)

**Figure 1:**
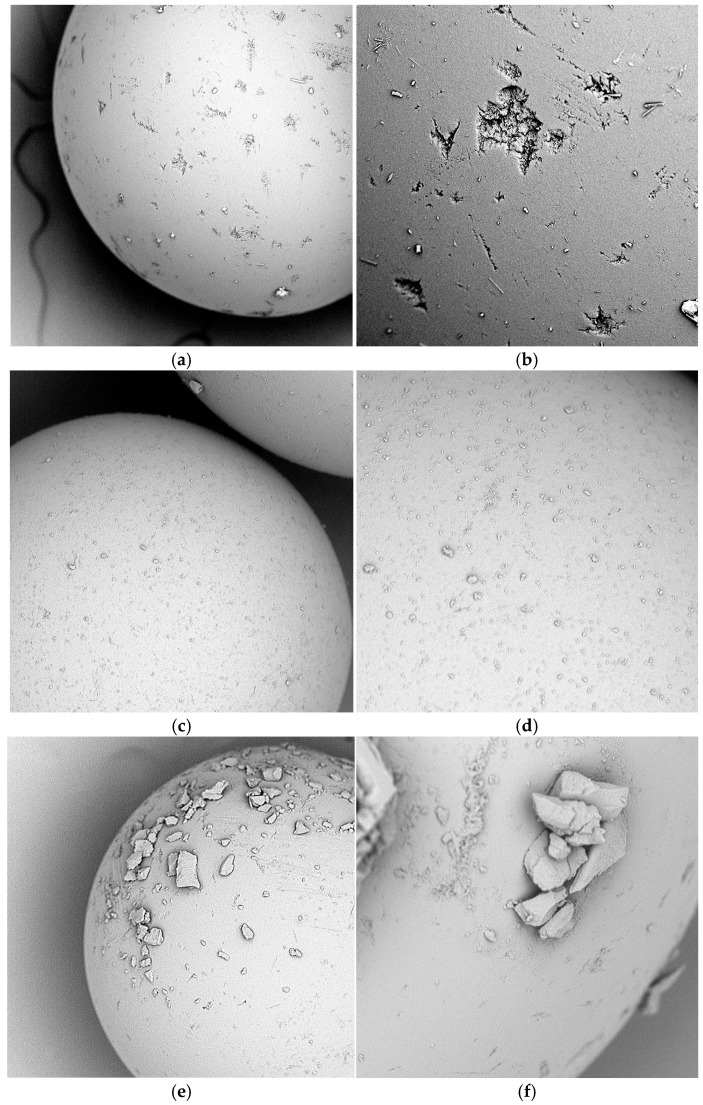
SEM pictures of: (**a**) Amberlite XAD-16 before impregnation mag. 500× (**b**) Amberlite XAD-16 before impregnation mag. 1000×; (**c**) Amberlite XAD-16-TBP (1:2) mag. 500×; (**d**) Amberlite XAD-16-TBP (1:2) mag. 1000×; (**e**) Amberlite XAD-16-TBP (1:3) mag. 500×; (**f**) Amberlite XAD-16-TBP (1:3) mag. 1000×.

The SEM analysis of the prepared impregnated sorbents: Amberlite XAD-16—TBP (1:2) and Amberlite XAD-16—TBP (1:3) was performed. Amberlite XAD-16 presented in Figure 1a,b is before the impregnation process. The surface of the sorbent was rough with visible indentations. The impregnated sorbent Amberlite XAD-16-TBP prepared at the ratio 1:2 is presented in Figure 1c,d. A much smoother surface of the sorbent coated with the extractant can be seen in the figures. Only negligible amounts of extractant are observed on the surface, indicating that the entire volume has been absorbed into the pores. Amberlite XAD-16-TBP impregnated at a ratio of 1:3 is shown in Figure 1e,f. As follows from the figures, much of the extractant remained on the surface. Too large a volume of TBP was used for impregnation; thus, it remained only in the sorbent pores. The figures below prove that the impregnation of the matrix was successful.

#### 2.1.2. Nitrogen Adsorption/Desorption Measurements

**Figure 2:**
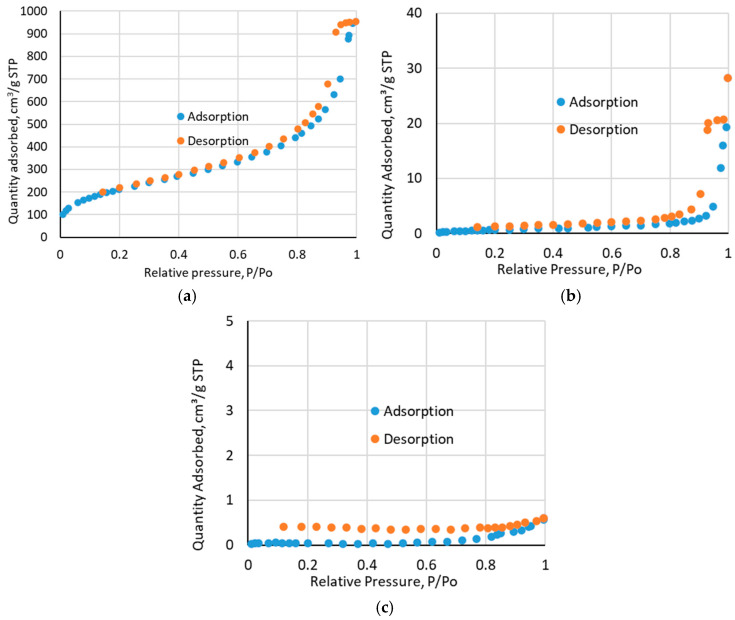
Isotherm linear plots for: (**a**) Amberlite XAD-16 before impregnation; (**b**) Amberlite XAD-16—TBP (1:2); (**c**) Amberlite XAD-16—TBP (1:3).

The isotherms plots are presented in Figure 2. Low-temperature nitrogen adsorption/desorption measurements showed that the BET surface area of the sorbent Amberlite XAD-16 was 777.10 m^2^/g and the pore size was 1.4755 cm^3^/g before impregnation. For impregnated Amberlite XAD-16—TBP (1:2), it decreased to 2.41 m^2^/g and 0.0436 cm^3^/g, respectively. The results for Amberlite XAD-16—TBP (1:3) showed that the BET surface area was 0.16 m^2^/g and the pore size was 0.000931 cm^3^/g, indicating that the sorbent pores are completely filled. It proved that the impregnation process has taken place.

#### 2.1.3. Fourier Transform Infrared Spectroscopy—Attenuated Total Reflectance (FTIR-ATR)

**Figure 3:**
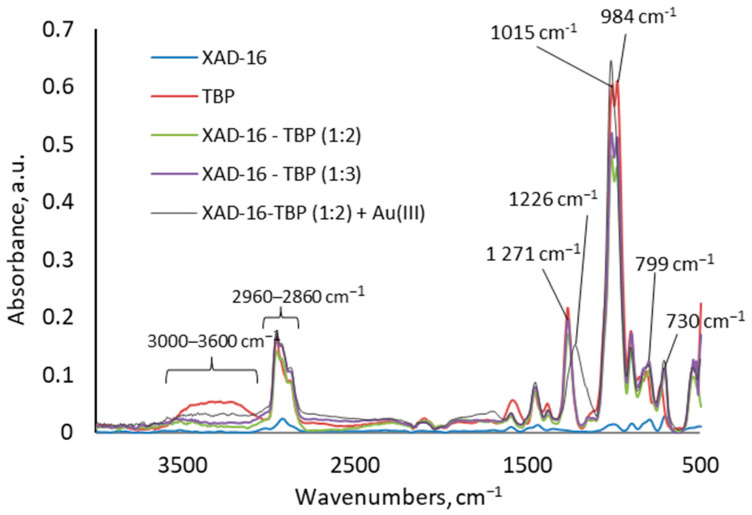
FTIR-ATR spectra for Amberlite XAD-16 before impregnation, the extractant TBP, and the impregnated sorbents: Amberlite XAD-16—TBP (1:2) and Amberlite XAD-16—TBP (1:3).

The FTIR-ATR spectra of TBP and Amberlite XAD-16 before and after impregnation with TBP are presented in Figure 3. The TBP contains P=O bonds at 1271 cm^−1^; these bonds do not occur in the Amberlite XAD-16 structure before impregnation [26]. After the impregnation process, the characteristic bond P=O is present in the Amberlite XAD-16—TBP (1:2 and 1:3) spectra at 1271 cm^−1^. Moreover, the intensities of these peaks are greater at a ratio 1:3 than at 1:2, indicating a higher concentration of TBP at a ratio of 1:3 than at 1:2. Similarly, the P-O-C stretching vibration bands can be observed at 984 and 1015 cm^−1^ in the TBP spectra. After impregnation of Amberlite XAD-16—TBP (1:2 and 1:3), these bands are present in the FTIR-ATR spectra and the intensity of these peaks is greater at a ratio 1:3 than at 1:2. Symmetric P-O-C vibrations appear in TBP and Amberlite XAD-16—TBP (1:2 and 1:3) spectra at 799 cm^−1^. The signal at 730 cm^−1^ can be assigned to the C-C-C skeletal vibrations. The stretching vibration signals C-H for the methyl and methylene groups in TBP structure appear in the region 2860–2960 cm^−1^. The C-H stretching vibration signals are observed in the Amberlite XAD-16 spectra at 2830–2930 cm^−1^. After impregnation, the C-H stretching vibration signals are more intensive than before impregnation. A broad signal in the range 3000–3600 cm^−1^ observed in the TBP spectra, is related to the O-H stretching vibrations of water molecules. The FTIR –ATR analysis confirms the effectiveness of the impregnation process.

### 2.2. Influence of Sorbent Mass, HCl Concentration, and Contact Time

The recovery percentage (R%) was calculated from the following equation:

(1) $$R\%=\frac{C}{C_{0}}\times 100\%$$

where *c* is the concentration of the sorbed precious metal ions (mg/L) and *c*_0_ is the initial concentration of precious metal ions (mg/L).

**Figure 4:**
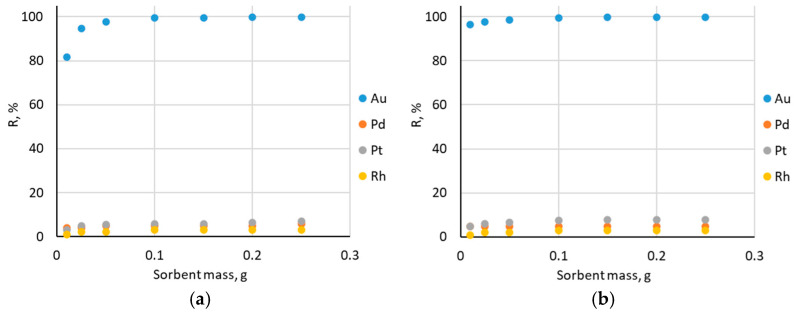
Dependence of recovery percentage on the mass of impregnated sorbents for: (**a**) Amberlite XAD-16—TBP (1:2) and (**b**) Amberlite XAD-16—TBP (1:3).

To determine the optimum sorbent mass used in the study, the effect of the impregnated sorbent mass on the precious metal ions sorption from the chloride solutions was investigated. This correlation is shown in the graph in Figure 4.

The amount of Au(III) ions removed from the solution at first increased slightly with the increasing sorbent mass for both Amberlite XAD-16—TBP (1:2) and Amberlite XAD-16—TBP (1:3). For the other elements—Pd(II), Pt(IV), and Rh(III)—the sorption was negligible. After crossing a sorbent mass of 0.1 g, the sorption of Au(III) ions was maintained at the same level. Increasing the sorbent mass did not result in more efficient removal of Au(III) ions from solution. Therefore, 0.1 g was considered to be the optimum sorbent mass used in the study.

**Figure 5:**
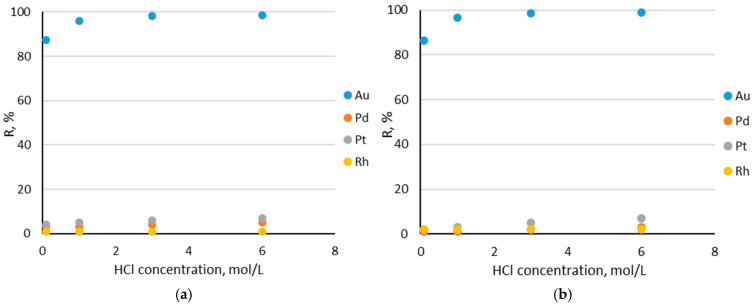
Dependence of precious metal ions sorption on HCl concentration (0.1, 1, 3, and 6 M): (**a**) Amberlite XAD-16—TBP (1:2) and (**b**) Amberlite XAD-16—TBP (1:3).

The effect of the HCl concentration on the recovery percentage of the precious metal ions were studied. The results of the Au(III), Pd(II), Pt(IV), and Rh(III) ions sorption in the HCl concentration range, i.e., 0.1, 1, 3, and 6 M, are presented in Figure 5.

The best efficiency was obtained for Au(III) ions. As follows from the graphs, the recovery percentage of the precious metal ions increases with the increasing HCl concentration. At 6 M HCl, the recovery percentage of the Au(III) ions was 98.5% for Amberlite XAD-16—TBP (1:2) and 98.8% for Amberlite XAD-16—TBP (1:3). The ions sorption of the other elements was very small and did not exceed 10%. This also proves the exceptional selectivity of the prepared sorbents, Amberlite XAD-16—TBP (1:2) and Amberlite XAD-16—TBP (1:3), towards Au(III) ions. The sorption of Au(III) ions is most efficient because the chloro-complexes formed by Au(III) have a much lower charge density than those of the other precious metals. The sorption of precious metals is affected by many factors, e.g., the size, the charge density, and the geometry of the precious metal chloro-complexes.

**Figure 6:**
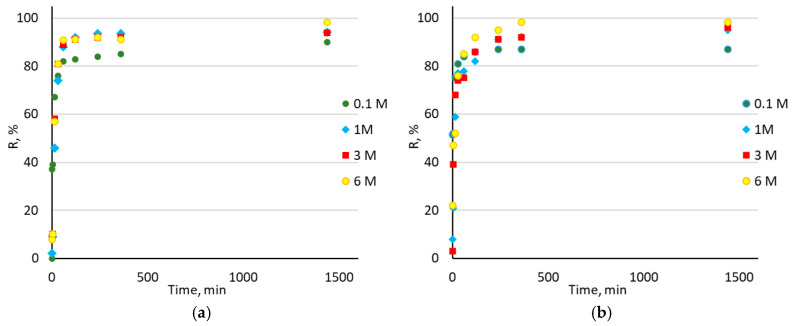
Dependence of Au(III) ions sorption on contact time (1, 5, 15, 30, 60, 120, 240, 360, and 1440 min) at HCl concentration range 0.1–6 M for: (**a**) Amberlite XAD-16—TBP (1:2) and (**b**) Amberlite XAD-16—TBP (1:3).

The effect of contact time on the sorption of Au(III) ions was also investigated and the results are shown in Figure 6. The results for the other metal ions (Pd(II), Pt(IV), and Rh(III)) are not presented in the graph, due to their insignificant sorption.

It is evident that the number of removed Au(III) ions increases with the time the sorbent is in contact with the solution. As mentioned above, the highest recovery percentage of Au(III) ions was obtained for the HCl concentration 6 M. At this acid concentration, sorption occurred rapidly—after only 60 min, more than 90% of all Au(III) ions were sorbed onto the sorbents, for both Amberlite XAD-16—TBP (1:2) and Amberlite XAD-16—TBP (1:3). After 24 h, the recovery percentage for Amberlite XAD-16—TBP (1:2) at 6 M HCl was 98.2%. Conversely, for Amberlite XAD-16—TBP (1:3), it was not much higher, being equal to 98.3%. The mechanism of association of the chloride Au(III) complex with TBP can be represented by the equation:

(2) $${AuCl}_{4}^{-}+H^{+}+TBP\to {HAuCl}_{4}\cdot TBP$$

TBP in the water undergoes ionic association, by which the TBP molecule is protonated by H^+^ (from the hydronium ion, H_3_O^+^), and thus, it can combine with a negatively charged Au(III) complex as the organo-metallic complex [27]. As follows from Equation (2), an increase in the concentration of H^+^ ions causes a shift of the equilibrium state to the right, and therefore, an increase in the concentration of HCl acid in the range of 0.1–6 M causes an increase in the R% value. It can be seen in Figure 3 that after sorption of Au(III) ions, the signal for stretching vibration of P=O bands is shifted to 1226 cm^−1^. The P=O group acts as a proton acceptor; therefore, the band is shifted towards lower frequencies. This is evidence that Au(III) chloro-complexes are sorbed by Amberlite XAD-16—TBP in the hydrochloric acid media.

### 2.3. Kinetic and Isotherm Studies

In order to study kinetics of the Au(III) ions sorption, four theoretical kinetic models were applied to analyze experimental results: pseudo-first-order, pseudo-second-order, Dunwald–Wagner, and intra-particle diffusion models. These kinetic models are most commonly used to describe the sorption of precious metal ions on various types of the sorbents [28,29,30,31]. The kinetic parameters were calculated based on the equations shown in Table 1.

The results of the calculations are presented in Table 2. For sorption of both Amberlite XAD-16—TBP (1:2) and Amberlite XAD-16—TBP (1:3), the greatest linear regression correlation coefficient was obtained for the PSO model being above 0.999, which is much higher than for the PFO. The largest sorption capacity of Au(III) ions was at 6 M HCl, and in the experiments, it was equal to 2.46 mg/g for Amberlite XAD-16—TBP (1:2) and 2.47 mg/g for Amberlite XAD-16—TBP (1:3). The sorption capacity values obtained with the PSO model resembled the greatest extent to the experimental results: 2.47 mg/g (Amberlite XAD-16—TBP (1:2)) and 2.49 mg/g (Amberlite XAD-16—TBP (1:3)). For both diffusion kinetic models, linear regression correlation coefficients were small, which indicated that the diffusion process is not mainly responsible for the sorption of Au(III) ions. The best fit to the PSO model is indicative of the chemisorption mechanism taking place between the TBP and the Au(III) chloro-complex. S. Jafari used TEMPO-oxidized nanofiber cellulose and the Au(III) adsorption kinetics were also best described by the PSO model [32].

For further analysis of Au(III) ion sorption on the impregnated sorbents, the experimental data were fitted to the three isotherm models: Langmuir, Freundlich, and Temkin. Equations of these isotherms and calculated parameters are shown in Table 3.

From the data presented in Table 3, it can be seen that the best fit was obtained for the Langmuir isotherm model. The Langmuir isotherm model indicates formation of a monolayer on the sorbent surface. The linear regression correlation coefficients were 0.9995 for Amberlite XAD-16—TBP (1:2) and 0.9996 for Amberlite XAD-16—TBP (1:3). The sorption of Au(III) ions on both impregnated sorbents was favorable, as evidenced by the value of the separation factor *R_L_* being in the range: 0 < *R_L_* < 1. The sorption capacities obtained in calculations for the Langmuir isotherm resembled to the greatest extent to those determined experimentally. In the experiments, the maximum sorption capacities were 145.93 mg/g for Amberlite XAD-16—TBP (1:2) and 147.64 mg/g for Amberlite XAD-16—TBP. The calculated values were 147.91 mg/g for Amberlite XAD-16—TBP (1:2) and 149.66 mg/g for Amberlite XAD-16—TBP (1:3). For comparison, M. Morcali et al. used commercially available L-214 and ARH adsorbents for gold recovery. In the study, the maximum adsorption capacities of 108.7 mg/g and 93.46 mg/g, respectively, were obtained [33].

### 2.4. Thermodynamic Studies

To investigate the effect of temperature on Au(III) ions sorption on Amberlite XAD-16—TBP (1:2) and Amberlite XAD-16—TBP (1:3), sorption studies were carried out at three different temperatures: 298, 303, and 313 K. Based on the obtained results, thermodynamic parameters, such as Gibbs energy change, Δ*G*°, entropy change, Δ*S*°, and enthalpy change, Δ*H*°, were determined. The equations and the values calculated from them are shown in Table 4.

The values of the Gibbs free energy change for sorption on both impregnated sorbents were negative, which means that the process was spontaneous. With the increasing temperature, the values decrease, which means that temperature inhibits the sorption process. Moreover, the enthalpy change values were negative, indicating that the occurring sorption of Au(III) ions on the prepared sorbents is an exothermic process. The negative values of entropy change indicate that the system is more ordered. In the study conducted by N. Sadeghi et al. the solvent extraction of Au(III) using TBP was performed. The thermodynamic results also indicated that gold recovery was an exothermic process [34].

### 2.5. Desorption Studies

**Figure 7:**
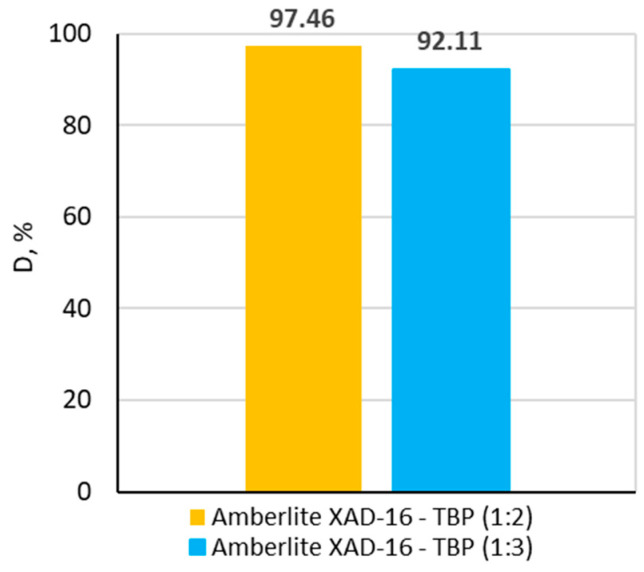
Results of Au(III) ions desorption with 1 M HCl/1 M TU for Amberlite XAD-16—TBP (1:2) and Amberlite XAD-16—TBP (1:3).

The possibility of reusing the impregnated sorbents was examined. The mixture of 1 M hydrochloric acid and 1 M thiourea was used as a desorbing solution. The evidence of effectiveness of using 1 M HCl and 1 M TU mixture for desorption of precious metal ions can be found in many papers [35,36]. The results of desorption studies can be seen in Figure 7.

For both impregnated sorbents, the number of desorbed Au(III) ions was very large. For Amberlite XAD-16—TBP (1:2), the desorption percentage was equal to 97.46%. Meanwhile, for Amberlite XAD-16—TBP (1:3), it was 92.11%. Thiourea forms strong gold cationic complexes, so it can easily replace chloride ligands in the Au(III) chloro-complexes. Such high values obtained in desorption studies demonstrate the prospective reuse of the newly developed impregnated sorbents: Amberlite XAD-16—TBP (1:2) and Amberlite XAD-16—TBP (1:3). The lower desorption percentage for Amberlite XAD-16—TBP (1:3) can be explained by the larger amount of TBP, which inhibits a contact between the thiourea molecules and the complexed Au(III) ions ${HAuCl}_{4}\cdot TBP$ present in the pores of the impregnated sorbent.

### 2.6. Sorption–Desorption Studies in the Real Leaching Solution

**Figure 8:**
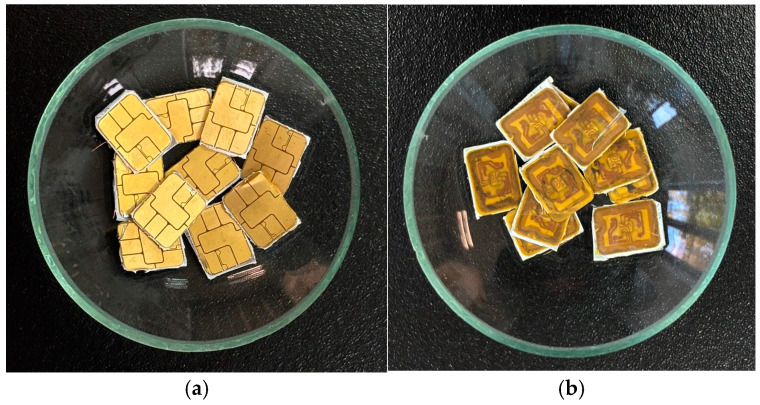
Chips from the smart cards: (**a**) before leaching; (**b**) after leaching.

The smart card chips were digested in the HCl/H_2_O_2_ mixture to perform studies in the real leaching solution. Before and after leaching, the cut-out chips are presented in Figure 8. Before leaching, the weight of the chips was 1.248 g, and after leaching, it was 0.981 g. Gold, together with other metals, passed into the solution. Table 5 shows the composition of the resulting solution.

**Figure 9:**
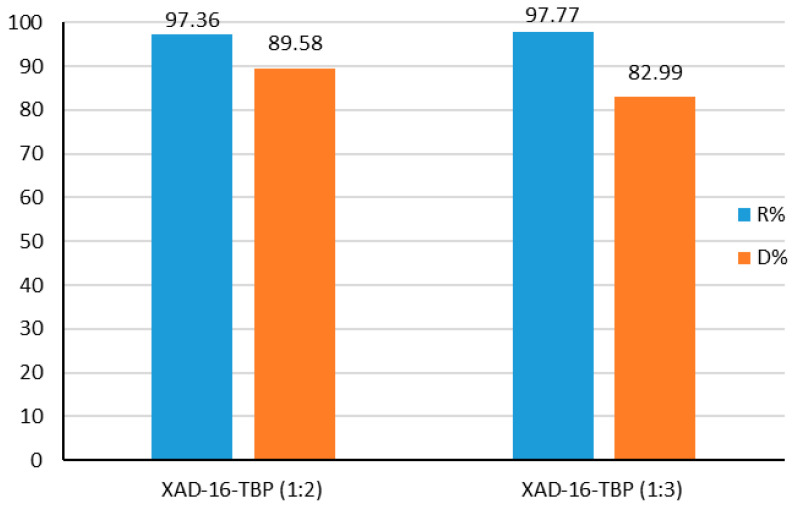
Recovery and desorption percentage of Au(III) ions for the real leching solution.

Amberlite XAD-16—TBP (1:2) and Amberlite XAD-16—TBP (1:3) were contacted with the resulting solution to study Au(III) ions sorption from the real leaching solution in the presence of the interfering elements, including macro-component (Cu(II) ions). The results of the sorption studies showed that Au(III) ions were removed from the solution with great efficiency. After 24 h min, 97.36% of Au(III) ions were sorbed on Amberlite XAD-16—TBP (1:2). For Amberlite XAD-16—TBP (1:3), the recovery percentage was 97.77%. The recovery percentage of Au(III) ions is presented in Figure 9. The results proved that the prepared sorbents are effective in Au(III) ion removal from a real leaching solution.

After sorption of Au(III) ions from the resulting solution, a desorption process was performed using 1 M HCl/1 M TU as a desorbing agent. The desorption percentage are presented in Figure 9. The results show high values of %D for the impregnated sorbents—89.58% for Amberlite XAD-16—TBP (1:2) and 82.99% for Amberlite XAD-16—TBP (1:3). It proved that the prepared sorbents can be useful in the recovery of Au(III) ions from the real leaching solutions.

## 3. Materials and Methods

### 3.1. Matrix

**Figure 10:**
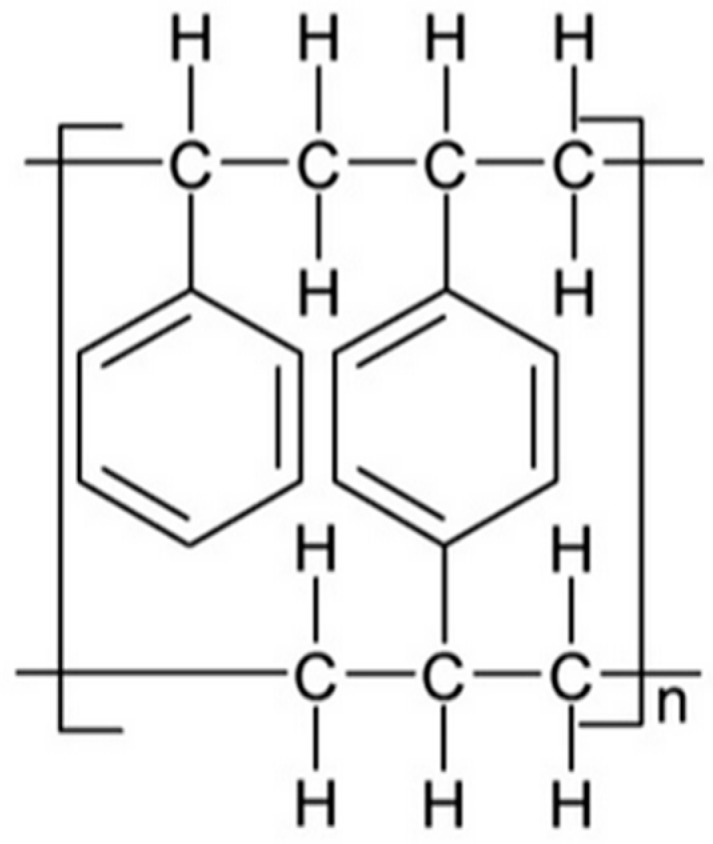
Structure of Amberlite XAD-16.

The resin used in the research was Amberlite XAD-16 (Rohm and Haas, Esslingen, Germany). This is a polymeric nonionic macroreticular resin whose surface area is 800 m^2^/g (according producer data sheet). It is in the form of white translucent beads with sizes: 0.56–0.71 mm and pore size of 200 Å. The structure of the matrix is presented in Figure 10.

### 3.2. Extractant

**Figure 11:**
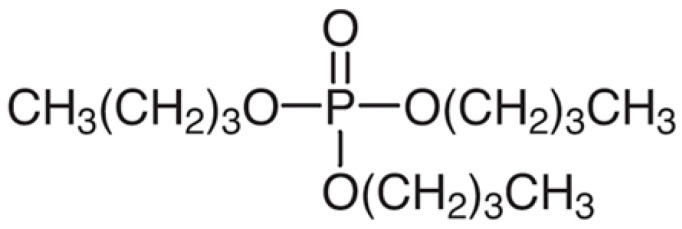
Structure of TBP.

Amberlite XAD-16 was impregnated with tributyl phosphate (C_12_H_27_O_4_P), commonly known as TBP (Fluka AG, Buchs, Switzerland). It is a colorless and odorless organophosphorus liquid used in a solvent extraction as extractant or as a plasticizer for cellulose esters. Its structure is given in Figure 11.

### 3.3. Precious Metals Solutions

In the research, HAuCl_4_, PdCl_2_, H_2_PtCl_6_ (POCH, Gliwice, Poland), and H_3_RhCl_6_ (ROMIL, Cambridge, UK) were used for preparation of precious metals solutions (10 mg/L of each element). Au(III), Pd(II), Pt(IV), and Rh(III) reference solutions (1000 mg/L of each element) supplied by ROMIL, Cambridge, UK was used to prepare standard solutions of precious metals for the ICP-OES analysis. The demineralized water (18.2 MΩ‧cm) used in the research was obtained from the Polwater DL2-150 system (Polwater, Cracow, Poland).

### 3.4. Method of Impregnation

The impregnation method used in the studies is called “warm impregnation”. It involves direct mixing the extractant and the matrix at the appropriate weight ratio without toxic organic solvents, such as chloroform, tetrahydrofuran, dichloromethane, hexane, pentane, etc. Mixing of the sorbent with the extractant takes place after heating the extractant to an optimum temperature that is lower than its boiling point. In the following experiment, Amberlite XAD-16 was mixed with TBP at a 1:2 and 1:3 weight ratio at 343 K. At a ratio of 1:1, the volume of extractant was insufficient to cover all sorbent beads.

### 3.5. Analysis of the Impregnated Sorbent

To confirm impregnation of the matrix, a scanning electron microscopy (Phenom World scanning electron microscope, Thermo Scientific, Waltham, MA, USA), Fourier transform infrared spectroscopy—attenuated total reflectance (Agilent Cary 630, Agilent Technologies, Santa Clara, CA, USA), and low-temperature (77 K) nitrogen adsorption/desorption measurements using an accelerated surface area and porosimetry system (analyzer ASAP 2045, Micromeritics, Norcross, GA, USA) were used in the study.

### 3.6. Influence of Sorbent Mass, HCl Concentration, and Contact Time

Au(III), Pd(II), Pt(IV), and Rh(III) solutions (10 mg/L of each element) at different HCl concentrations, i.e., 0.1, 1, 3, and 6 M, were prepared. Moreover, 0.2 g of each sorbent was contacted with 50 mL of the solutions and the samples were shaken in the laboratory shaker Elpin 358A (Elpin, Lubawa, Poland). After a specific time (1, 5, 15, 30, 60, 120, 240, 360, and 1440 min), the samples were taken.

Influence of the sorbent mass was been studied. The impregnated sorbents masses of 0.01, 0.025, 0.05, 0.1, 0.15, 0.2, and 0.25 g were weighed and contacted with 25 mL of precious metals solution (10 mg/L of Au(III), Pd(II), Pt(IV), and Rh(III)) at 6 M HCl. Then, the samples were shaken for 24 h in a laboratory shaker.

To study the influence of HCl concentration on Au(III), Pd(II), Pt(IV), and Rh(III) ions sorption, the determined optimum sorbent mass: 0.1 g was contacted with 25 mL of the solutions containing 10 mg/L of each element at different HCl concentrations (0.1, 1, 3, and 6 M) and the samples were shaken in the laboratory shaker for 24 h.

Precious metals concentrations were determined by inductively coupled plasma-optical emission spectrometry Varian 720 ES ICP-OES (Varian, Melbourne, Australia).

### 3.7. Isotherms

In total, 0.1 g of the impregnated sorbents was brought in contact with 25 mL of Au(III) solutions with the following concentrations: 10, 25, 50, 100, 250, 500, 750, and 1000 mg/L, at 6 M HCl. The samples were shaken for 1440 min in a laboratory shaker. Au(III) concentrations after sorption were determined by ICP-OES. 

### 3.8. Thermodynamics

In total, 0.1 g of the impregnated sorbents was equilibrated with 25 mL of a precious metal (10 mg/L of Au(III), Pd(II), Pt(IV), and Rh(III)) solution. The samples were shaken for 24 h in the laboratory shaker at the temperatures: 293, 303, and 333 K. The concentrations after the sorption were determined by ICP-OES.

### 3.9. Desorption Studies

For desorption studies, 0.1 g of impregnated sorbents loaded with Au(III) ions was contacted with 25 mL of a desorbing agent—a mixture of 1 M HCl and 1 M thiourea. Desorption process was performed for 24 h in laboratory shaker. After sample collection, the concentration of Au(III) ions in the solution was determined by ICP-OES.

### 3.10. Sorption–Desorption Studies in the Real Leaching Solution

In the following experiment, chips were cut out from the smart cards for digestion with the HCl/H_2_O_2_ mixture. The cut-out chips were treated with 25 mL concentrated HCl, and then 25 mL H_2_O_2_ was added in portions. The prepared solution was heated for 3 h at 333 K. The samples were taken after cooling down the mixture. The concentrations of metals contained in the solution were determined by inductively coupled plasma-optical emission spectrometry. The real leaching solution was used in the Au(III) ions sorption studies. Moreover, 10 mL of the real leaching solution was brought in contact with 0.04 g of the prepared impregnated sorbents. The samples were taken after shaking them for 1440 min in the laboratory shaker. The concentrations of the Au(III) ions were analyzed by ICP-OES.

After the sorption studies, 0.04 g of the loaded sorbent was contacted with 10 mL of the 1 M HCl/1 M TU mixture to perform the desorption process. The samples were shaken in the laboratory shaker for 1440 min and then determined by ICP-OES.

## 4. Conclusions

Based on the study, it can be concluded that the prepared Amberlite XAD-16 sorbents impregnated with TBP can be an effective material for the recovery of Au(III) ions from chloride solutions formed after leaching of electronic waste (gold chips from smart cards). Au(III) ions were also effectively removed from the real leaching solution with the efficiency: 97.36% for Amberlite XAD-16—TBP (1:2) and 97.77% for Amberlite XAD-16—TBP (1:3). The sorption studies indicated that when using 25 mL of precious metal solution the optimum sorbent mass was 0.1 g. The best sorption efficiency was obtained at the 6 M HCl concentration. An increase in the acid concentration in the range of 0.1–6 M HCl causes an increase in the sorption capacity of Au(III) ions on the impregnated sorbent. From the kinetic study, it can be concluded that the best kinetic model describing the sorption of Au(III) ions was the PSO model (R^2^ value was above 0.999 for both prepared sorbents). The best-fitting isotherm was the Langmuir isotherm—the sorption capacities determined during the calculations at 6 M HCl were 147.91 mg/g for Amberlite XAD-16—TBP (1:2) and 149.66 mg/g for Amberlite XAD-16—TBP (1:3). These values were the closest to the experimental ones, i.e., 145.93 mg/g and 147.64 mg/g, respectively. The determined free Gibbs energy, enthalpy change, and entropy change values were negative, indicating that the process was spontaneous, exothermic, and proceeded with increasing order in the system. Using the mixture of 1 M HCl/1 M TU, Au(III) ions can be effectively removed from the sorbent. The results for the model chloride solutions (6 M HCl) for the sorbents prepared at the ratio 1:2 and 1:3 were as follows: 97.46% and 92.11%. Conversely, for the real leaching solution, they were: 89.58% and 82.99%. The obtained sorbent is very selective towards Au(III) ions in the strong acidic solution containing copper(II) ions as a macro-element. After suitable optimization, the prepared sorbents can be used for the industrial WEEE recycling process and the recovery of Au(III) from the real solutions formed after leaching, e.g., RAM modules, central processing units, printed circuit boards, contacts, or hard disks drives, which will be presented in future publications.

## Figures and Tables

**Table 1:** Equations used to describe Au(III) ions sorption kinetics.

Kinetic Model	Equation	Eq. Number
Pseudo-first-order	$\ln\left(q_{e}-q_{t}\right)=lnq_{e}-k_{1}\times t$, where *q_e_*—the equilibrium adsorption capacity (mg/g); *q_t_*—the adsorption capacity at a specific time (mg/g); *k*_1_—the pseudo-first order speed constant (min^−1^); *t*—the contact time (min)	(3)
Pseudo-second-order	$\frac{t}{q_{t}}=\frac{1}{k_{2\cdot }q_{e}^{2}}+\frac{1}{q_{e}}\times t$, where *q_e_*—the equilibrium absorption capacity (mg/g); *q_t_*—the adsorption capacity at a specific time (mg/g); *k*_2_—the pseudo-second order speed constant (g/mg‧min); *t*—the contact time (min)	(4)
Intra-particle diffusion	$q_{t}=k_{ip}\times t^{0.5}+C$, where *q_t_*—the adsorption capacity at a specific time (mg/g); *k_ip_*—the intra-particle diffusion rate constant (mg/g·min^0.5^); *t*—the contact time; *C*—the intercept (mg/g)	(5)
Dunwald–Wagner	$\log\left(1-{\left(\frac{q_{t}}{q_{e}}\right)}^{2}\right)=-\frac{k}{2.303}t$, where *q_e_*—the equilibrium absorption capacity (mg/g); *q_t_*—the adsorption capacity at a specific time (mg/g); *k*—the diffusion rate constant (min^−1^); *t*—the contact time (min);	(6)

**Table 2:** Kinetic parameters for the Au(III) ions sorption on the impregnated sorbents: Amberlite XAD-16—TBP (1:2) and Amberlite XAD-16—TBP (1:3).

**Amberlite XAD-16—TBP (1:2)**							
**PFO**				**PSO**			**Experimental**
HCl (M)	*k*_1_ (1/min)	*q_e_* (mg/g)	R^2^	*k*_2_ (g/mg‧min)	*q_e_* (mg/g)	R^2^	*q_t_* (mg/g)
0.1	0.0073	0.98	0.6232	0.0221	2.23	0.9995	2.25
1	0.0140	0.95	0.8394	0.0233	2.38	0.9991	2.34
3	0.0089	0.79	0.6262	0.0322	2.39	0.9994	2.37
6	0.0079	0.90	0.6746	0.0333	2.47	0.9998	2.46
**Dunwald–Wagner**				**Intra-Particle Diffusion**			
HCl (M)	*K* (min^−1^)		R^2^	*k* (mg/g‧min^0.5^)	*C* (mg/g)	R^2^	
0.1	0.0060		0.6626	0.0500	0.9352	0.4628	
1	0.0127		0.8793	0.0503	1.0839	0.4038	
3	0.0077		0.6666	0.0476	1.1653	0.3914	
6	0.0068		0.7313	0.0475	1.2269	0.4062	
**Amberlite XAD-16—TBP (1:3)**							
**PFO**				**PSO**			**Experimental**
HCl (M)	*k*_1_ (1/min)	*q_t_* (mg/g)	R^2^	*k*_2_ (g/mg‧min)	*q_e_* (mg/g)	R^2^	*q_t_* (mg/g)
0.1	0.0113	1.12	0.8135	0.0271	2.30	0.9995	2.28
1	0.0104	1.34	0.9346	0.0275	2.38	0.9999	2.36
3	0.0073	0.95	0.8492	0.0384	2.41	0.9999	2.40
6	0.0135	1.23	0.9463	0.0481	2.49	0.9999	2.47
**Dunwald–Wagner**				**Intra-Particle Diffusion**			
HCl (M)	*K* (min^−1^)		R^2^	*k* (mg/g‧min^0.5^)	*C* (mg/g)	R^2^	
0.1	0.0099		0.8468	0.0513	0.9529	0.4810	
1	0.0092		0.9665	0.0500	1.0263	0.5311	
3	0.0065		0.8903	0.0379	1.3665	0.5321	
6	0.0126		0.9550	0.0424	1.3627	0.5131	

**Table 3:** Langmuir, Freundlich, and Temkin isotherm models equations, constants, and determination coefficients for Au(III) ions sorption on the impregnated sorbents.

Isotherm Model	Equation		Parameter	Amberlite XAD-16—TBP (1:2)	Amberlite XAD-16—TBP (1:3)
Langmuir	$q_{e}=\frac{{q_{m}K}_{L}C_{e}}{1+K_{L}C_{e}}$	(7)	*q_m_*	147.91	149.66
			*K_L_*	0.1380	0.1434
	$R_{L}=\frac{1}{1+K_{L}C_{0}}$	(8)	*R_L_*	0.0962	0.0936
			R^2^	0.9995	0.9996
Freundlich	$q_{e}=K_{F}{C_{e}}^{\frac{1}{n}}$	(9)	*K_F_*	15.52	15.97
			*n*	2.14	2.14
			R^2^	0.7942	0.7941
Temkin	$\frac{q_{e}}{q_{m}}=\frac{RT}{b_{T}}lnK_{T}C_{e}$	(10)	*K_T_*	5.06	5.53
			*b_T_*	17.86	18.05
			R^2^	0.9292	0.9201

where *q_e_*—the amount of adsorbate (mg/g); *q_m_*—the maximum adsorption capacity (mg/g); *K_L_*, *K_F_*, *K_T_*—the constant related to the adsorption capacity; *R_L_*—the separation factor related to Langmuir isotherm; *C_e_*—the equilibrium concentration in the solution; *n*—the adsorption intensity; *R*—the universal gas constant (8.314 J/K‧mol); *T*—the temperature (K); *b_T_*—the Temkin constant related to the heat of adsorption (kJ/mol).

**Table 4:** Thermodynamic parameters for Au(III) ions sorption on the impregnated sorbents: Amberlite XAD-16—TBP (1:2) and Amberlite XAD-16—TBP (1:3).

Equation		Thermodynamic Parameter	Temperature	Amberlite XAD-16—TBP (1:2)	Amberlite XAD-16—TBP (1:3)
$∆G{}^{\circ}=–RTlnK_{D}$	(11)	Δ*G*° [kJ/mol]	293 K	−6.10	−6.29
			303 K	−5.84	−6.05
			313 K	−5.72	−5.96
$ln\left(K_{D}\right)=-\frac{∆H{}^{\circ}}{RT}+\frac{∆S{}^{\circ}}{R}$	(12)	Δ*S*° [J/mol]		−19.30	−17.10
		Δ*H*° [kJ/mol]		−11.74	−11.29

where ∆G°—the free Gibbs energy (kJ/mol); *R*—the universal gas constant (8.314 J/K‧mol); *T*—the temperature (K); *K_D_*—the distribution coefficient (L/g); Δ*H*°—the enthalpy change (kJ/mol); Δ*S*°—the entropy change (J/mol).

**Table 5:** Composition of the solution after leaching the chips from smart cards.

Element	Au(III)	Cu(II)	Ni(II)	Ag(I)
Concentration (mg/L)	4.93	3991.70	833.33	0.72

## Data Availability

The data are included in the article and available upon request.
